# A Medical Army Reserve

**Published:** 1900-12-22

**Authors:** 


### The Hospital

A Journal of

The Medical Sciences and Hospital Administration.

Vol. XXIX.—No. 743.] Edited by Sir Henry Burdett, K.C.B., and Solomon C. Smith, M.D., M.R.C.P. [December 22, 1900

### A Medical Army Reserve.

With the return of Lord Roberts to England it is reasonable to hope that the grave question of how best to remedy the defects in our military system which have been disclosed by the war, will at once receive the attention of all who will have any share in the preparation of measures for the consideration of the Legislature. It is equally important that this question should also receive consideration from the public; because, if past experience teaches anything, it teaches that existing abuses have to a large extent been the outcome of what are called "social" considerations, and that they will be supported, and that everything tending towards a reform of them will be opposed, by a plutocracy of very great influence and of very wide ramifications. Many of the objectionable conditions which have been rendered manifest during the last twelve months were at least equally manifest during the continuance of the Crimean war; and, then as now, the approach of peace was hailed as an event which would afford the nation an opportunity of setting its house in order. How completely the opportunity was missed or neglected, and how soon the attention of the public was diverted from the Army by the paltry squabbles of professional politicians, need hardly be told to-day, when the results of the neglect have carried sorrow and loss into so many homes; but the tendency of history is to repeat itself, and it behoves us so to use our experience as to guard, if possible, against a recurrence of conditions the past prevalence of which we have so much reason to deplore. Assuming, as we may fairly assume, that the Commander-in-Chief and the Secretary of State for War are in earnest, it is none the less imperative, if any good is to be done, that they should be thoroughly supported by the whole weight of public opinion.

The conditions which have existed in the Army for many years have produced among innumerable other evils an ignorant contempt for the health of the soldier, which has been followed, as a matter of course, by a large measure of neglect of the conditions by which alone that health can be secured. The medical department has been scandalously undermanned, even with reference to the requirements of peace, so that its officers have been deprived alike of ordinary relaxation and of opportunities of study; and it is of course utterly inadequate, numerically speaking, for the demands of war. Even if it were so enlarged as to provide for all ordinary requirements the numerical inadequacy in time of war would remain; and it is highly desirable that some permanent provision should be made for promptly raising the ranks of the department to fighting strength in time of need. We believe that this might be done without the smallest difficulty by an appeal to the patriotism of the profession, and by the concession of a few privileges of a kind calculated to render membership of an Army Medical Reserve somewhat of a distinction. An opportunity might be given to every young medical man who had obtained his qualifications, within proper limits of time, to enrol himself for a definite period —say, of five or ten years, according to the number of men available—in a Medical Reserve Corps to be employed only in time of war, but to be afforded opportunities, by short periods of camp training, of learning how the business of doctoring in war time is conducted. Such training might be arranged for at Aldershot, or other camps of instruction, or with Militia regiments; and there are probably not many practitioners whose engagements during the first few years of professional life would place any serious difficulties in the way of fulfilling such requirements as membership of a Reserve would demand from them. During the present war grave inconvenience, and even serious loss, has been occasioned to many well-established men who were surgeons to Militia regiments and who, after having for some years regarded their appointments chiefly in the light of occasions for pleasant autumnal outings, have been suddenly forced to recognise their binding nature, and perhaps to proceed to South Africa, in some cases to lose their health or even their lives there. Against such undesirable displacement of men of middle age the suggested Reserve would afford ample provision; and it is impossible to doubt that it might be both formed and kept together if the authorities of the War Office would not only cease from being obstructive, but would render assistance by conferring a few small privileges, such as the right to wear uniform on public occasions, to men who were willing, in return, to enter into binding engagements to keep themselves in a state of readiness to serve their country, whenever or wherever their services might be required.

